# Protein Aggregation Increases with Age

**DOI:** 10.1371/journal.pbio.1000449

**Published:** 2010-08-10

**Authors:** Rachel Jones

**Affiliations:** Freelance Science Writer and Editor, Welwyn, Hertfordshire, United Kingdom

**Figure pbio-1000449-g001:**
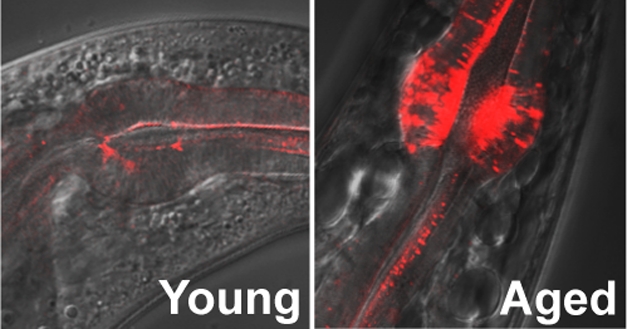
Several hundred proteins became highly insoluble with age in the worm. One of the proteins detected by mass spectrometry, KIN-19, shown in the figure, forms aggregates in the pharynx with age.

[Fig pbio-1000449-g001]Aggregation (“clumping together”) of certain cellular proteins is a common feature of a variety of diseases including neurodegenerative conditions such as Parkinson's, Alzheimer's, and Huntington's diseases. Many of these conditions are more prevalent in old age, but the changes that cause increased aggregation of these disease proteins are not well understood.

A new study by Cynthia Kenyon and colleagues shows that aging in the nematode *Caenorhabditis elegans* is associated with increased aggregation of a large number of proteins, even in the apparent absence of disease. Many of these proteins are encoded by genes that influence lifespan or aggregation of proteins with repeated polyglutamine sequences (the aggregation motif involved in Huntington's disease and other conditions), suggesting that an aging-associated increase in protein aggregation might affect both lifespan and neurodegeneration.

Because aggregated proteins are insoluble, the authors used a systematic proteomics approach to search for proteins that became more insoluble with age. They found several hundred insoluble proteins, many of which were present in greater amounts in older worms than in younger ones. When the authors compared different strains of worms, they found that rather than a general increase in protein insolubility, a specific subset of proteins consistently became more insoluble with age.

To confirm the association between insolubility and aggregation, the authors created worm lines in which specific proteins from the set that became more insoluble with age were tagged with fluorescent markers. As expected, these proteins formed aggregates in older animals. Closer studies showed that different proteins tended to form aggregates in a variety of tissues and subcellular compartments.

In younger animals, protein aggregation is kept in check by regulatory processes that control protein homeostasis, however, these processes seem to become less efficient with age. This might explain both the increased incidence of protein-aggregation diseases in old age and the propensity for a large subset of proteins to become more insoluble with age. To investigate the association of aging with protein aggregation, the authors looked at worms bearing a mutation in the insulin/IGF-1 (insulin-like growth factor 1) signalling pathway that doubles the lifespan of the animals and also delays progression in *C. elegans* models of protein aggregation diseases. Although young animals with this mutation showed the same levels of insoluble proteins as young wild-type worms did, there was a much smaller increase in insoluble proteins in older animals with the mutation.

When the authors looked more closely at the patterns of protein insolubility and the expression of proteins prone to insolubility in these long-lived animals, they found that certain aggregation-prone proteins were down-regulated, apparently by a reduction in gene transcription. The mutation in the insulin/IGF-1 signalling pathway, therefore, seems both to promote the solubility of proteins and also to reduce the transcription of certain aggregation-prone proteins.

One of the authors' findings has a particular bearing on neurodegenerative disease. In worm disease models, expression of polyglutamine-repeat proteins in the muscle causes progressive, age-related paralysis. When the authors expressed one of their aggregation-prone proteins—the kinase KIN-19—in the muscle alongside the polyglutamine-repeat protein Q35, the worms became paralysed earlier, even though the aggregation of Q35 itself did not increase and despite the fact that aggregation of KIN-19 alone in the muscle does not cause paralysis. This finding implies that age-related normal protein aggregation can increase the severity of disorders caused by aggregation of disease-related proteins.

When the authors compared the aggregation-prone set of proteins with the *C. elegans* proteome as a whole, they found that proteins whose inhibition can increase lifespan in worms were over-represented among the aggregation-prone proteins identified by the study. Aggregation itself might therefore limit lifespan by having negative effects on animals.

Compared with the whole proteome, the aggregation-prone set of proteins also had specific structural features. Certain amino-acid residues were over-represented in aggregation-prone proteins, and they were more likely to contain a secondary structure called a β-sheet. This is also found in certain disease-associated proteins, most notably β-amyloid, which forms aggregates in several human diseases including Alzheimer's disease.

Finally, the authors compared their list of inherent aggregation-prone proteins with lists of proteins typically found alongside disease proteins such as β-amyloid in aggregates in human disease. They found homologues of many proteins present at low levels in human disease aggregates become insoluble in worms. The inherent aggregation of these proteins might therefore affect the pathological processes involved in these diseases.

The finding that aging itself can increase the insolubility and aggregation of many proteins that are not implicated in disease raises many issues for further research into both aging and disease mechanisms. Kenyon and colleagues suggest that such research might lead to the identification of new therapeutic avenues, as well as to a greater understanding of how protein aggregation influences cellular and pathological processes.

**David DC, Ollikainen N, Trinidad JC, Cary MP, Burlingame AL, et al. (2010) Widespread Protein Aggregation as an Inherent Part of Aging in** ***C. elegans*** **. doi:10.1371/journal.pbio.1000450**

